# Central Dental Association of Northern New Jersey

**Published:** 1883-08

**Authors:** James G. Palmer

**Affiliations:** Sec’y.


### CENTRAL DENTAL ASSOCIATION OF NORTHERN NEW JERSEY.

A regular meeting of this Society was held at the office of Dr. Worthington Pinney, 72 Park Place, Newark, N. J., June 28, 1883. The President, Dr. F. A. Levy, occupied the chair. After the transaction of the usual routine business, Dr. Charles A. Meeker, of Newark, read the following paper:

### PHOSPHORUS IN THE FORM OF ACID PHOSPHATES.

Phosphorus was discovered two hundred and fourteen years ago by the alchemist Brandt, of Hamburg, and since that time has retained a position in the materia medica of all civilized countries. The present process of manufacture consists in digesting clean broken bones in dilute muriatic acid, thus forming chloride of calcium and acid phosphate of lime, the residual bone cartilage remaining undissolved. The solution is then evaporated, when the acid phosphate crystalizes. It is then dried, mixed with half its weight of charcoal, and distilled from clay cylinders, one hundred pounds of clean bones yielding six pounds of phosphorus.

Coincident with its discovery it was found to act as a powerful general stimulant in small doses, in large doses as a violent irritant poison. Its physiological action is particularly upon the kidneys and genital organs, producing excitation and diuresis. It is also considered applicable in cases of prostration of the vital powers. The many published formulas in the medical journals in which this remedy forms an important adjunct, and the many advertisements of patent medicines where it is likewise used, testify to the esteem in which this remedy is held by the practitioners of all schools. About ten years since I commenced its use in the form of the lacto-phosphate of lime, and perfected the following formula:

℞ Phosphate of Lime (fresh)—ʒ vi.

Aqua—℥ iv.

Muriatic Acid—℥ 1.

M.

This mixture will give a clear solution. Add aqua ammonia to precipitate, and draw off the liquor by the aid of a siphon, and so continue by refilling with water and siphoning off until all trace of alkali is gone. Add to your magma pure concentrated lactic acid 1½ *f* ℥, sugar 10½ ℥, orange flower water, 1 *f* ℥, and water to complete 10½ *f* ℥. This makes a very beautiful clear syrup, exceedingly pleasant to the taste, which I prescribed in a number of cases for defective nutrition, with good results, but was compelled to discontinue its use because of inability to keep it in warm weather, the making in small quantities causing a loss of time.

You all know that on several occasions at our meetings, I have called your attention to the employment of the Acid Phosphates, which I have now used nearly two years, and for the dental practitioner's purpose, I consider it the best way to administer phosphorus.

There is a significant proverb, "without phosphorus there is no thought." The fluid interior of the red corpuscles contains acid phosphate of potassa, the serum phosphate of soda, the juice of the muscles acid phosphate, the brain phosphoric acid. In pregnant women the phosphates disappear almost entirely from the urine and secretions, while in the pelvis and on the inner surface of the parietes of the skull is formed a deposition of bony matter supposed to contain a large amount of phosphates, which disappear in the later stages of pregnancy. It is probable that they are absorbed and utilized in the formation of the fœtal skeleton. When there is previous mal-nutrition or an æenemic state, with an insufficiency of the phosphates stored for the growing fœtus, there is a natural craving of the appetite for foods or minerals containing them, as indicated by the ravenous appetite for these foods, some finding it hard to withstand the temptation to eat the freshly made mortar used during the erection of a building, some having an inordinate craving for slate pencils, plaster of paris, marble, lime, etc., thus showing this appetite to be nature's demand for this element of life.

Blacke, of Paris, in order to obtain a correct knowledge of the action of the lime salts of nutrition, carefully submitted a pigeon to the test of taking food which was almost entirely deprived of the phosphates. It soon lost all its liveliness, its appetite, and a notable portion of its weight, while it excreted more phosphates than it absorbed, the muscular and fibrous tissues diminishing as rapidly as the skeleton.

The addition of phosphates to its ordinary food soon brought it back to its normal condition.

I regret that I have not with me this evening my tabulated statement of cases in which I have used the acid phosphates, and seen the benefits derived from them. Want of time has prevented, but I will give them at some future meeting.

My success has been most marked in children whose teeth, when about half erupted (more especially the permanent incisors) show an arrest of growth, deep furrows extending in part or wholly across the labial surfaces, corrugated cutting edges, an entire or partial denudation, large white or brown spots upon the surface, enamel soft and crumbling, rapid wearing down of the incising edges by attrition and occlusion, through softness of texture. Most of these appearances practitioners will observe in children of from 6 to 12 years of age, and in females about the age of puberty. I have seen good results following the use of this remedy in pregnant women with aching teeth.

The discussion which followed the reading of the paper was quite general, but not of interest sufficient to repay publication.

Dr. R. M. Sanger, of East Orange, then read the following paper upon

CHEOPLASTRY.

GENTLEMEN:

I do not apologise for addressing you on a subject which has neither the claim of novelty or originality, nor am I at this moment enabled to place before you any illustration or specimens to render my claim on your attention greater, but so confident am I of the immense practical value of my subject-matter that I have perfect confidence in gaining eventually your interest and gratitude for directing your attention to Cheoplastry. It is, indeed, a very old friend, so old that it might be great-grandfather to some of us, though we all claim a kind of relationship or acquaintance with it. I believe there are many like myself, who, for years of their professional life possessed less even than a nodding intimacy. Now that I am thoroughly familiar, I can only regret that I have been so long in ignorance of such a valuable, simple, and unerring system of dental prosthesis, and sincerely desire to give all who are unfamiliar with its merits confidence in adopting it.

About twenty-five years ago Dr. Blandy applied the term Cheoplastry to a system of constructing artificial dentures by means analogous to the vulcanite process, only in place of rubber an alloy of tin was used for the base. A full description of this and similar processes will be found in "Harris' Principles and Practice." It is easy to understand that the advent of rubber completely eclipsed Dr. Blandy's invention—for I believe he was the inventor—and it is probable that, like an over-zealous witness, he spoilt his case by saying and claiming too much, and proposing a too universal use for it. The system may be familiar to you as "Weston's Metal," or "Reese's Cast Gold Alloy," the means of manipulation being similar in all, and the most important ingredient of their metals being pure tin, which is possibly injured by the addition of the noble metals. Dr. Reese, in conjunction with the venerable Atkinson, has without doubt devoted much time, ability and energy to the producing of the present artistic results; but whether his metal possesses the necessary strength for use in many partial cases, or is as universally applicable as is claimed, I am not prepared to say; but, in all lower cases, whether partial or entire, I unhesitatingly aver that the Cheoplastic system surpasses any other at present within our knowledge. I do not even except the "Allen," or "Continuous Gum" work, though without doubt we may succeed with that where we fail with rubber. The most limited in experience are familiar with the patients "who can't manage their lower set, anyhow." They are early in your office, and the last to leave at night, and I have heard of their coming in the middle of the night. "It won't stay in its place," and truly that is a distressing defect. Of course I here refer only to full sets. Weighted rubber rarely mitigates the evil. Spiral springs do not in all cases succeed, while they possess so many objectionable qualities that few dentists care to have recourse to them, and fewer patients to submit to wearing them; but the cast metal base will at once meet the requirements of such conditions. The patient immediately seems to receive an inspiration of confidence; the denture remains firm and steady; no inflammation or excoriation is produced, or if it is, relief is readily afforded. Patients sometimes remark that they feel as if they had something in their mouth, but the same would apply to eating candy. Within the past month I have made successful dentures for two old ladies, whose cases were considered hopeless in several first-class offices, and would have been in my own, before adopting this method. The patients themselves are emotional in their expressions of surprise and gratitude at the successful result, affording them full capacity for mastication. I regret that it is not in my power to introduce these two cases to your personal notice, but unfortunately it is not. The modus operandi is at once simple and positive, much more so than rubber or celluloid. A minor advantage that I will here mention is the security you have of not checking gum blocks. Your case will come from the flask exactly as you have manipulated it in the wax, and by the display of only ordinary skill; little or no time will be required to give a high finish, which may be enhanced by electro gilding, though the metal itself will retain a color in the mouth equal to the best known amalgam.

To give an example of its general capabilities I have here the commonest of glass bottles, in which a casting has been made. It is unfractured, nor is any sign of leakage shown, though it has been saturated with dyed alcohol and strong acids. Comment thereon is unnecessary. The metal used is an alloy of tin, prepared by my assistant, Dr. Lawrence Vanderpant, who has spent many years in experimenting. I do not deem it necessary to trespass longer on your time and patience, except to state that I believe that the cheoplastic system, whether by means of Weston's, Reese's or any suitable metal (which, by the way, will never be one of the nobler metals), will be to the practitioner of dental prosthesis as essential as plastic fillings or the rubber dam to the operator.

Dr. Sanger's paper was discussed by some of the older members, the debate being mainly confined to the best mode of taking impressions.

Dr. E. H. Bunting, Sr., on behalf of the Society, and as a tribute of respect, then presented to Dr. W. P. Richards, who had but lately taken upon himself the vows of matrimony, a handsome clock. The presentation remarks of Dr. Bunting and the reception speech of Dr. Richards were happily expressed and warmly received.

The Society then adjourned, to meet in September at the office of Dr. L. C. G. Watkins, in Montclair.

James G. Palmer, Sec'y.

